# Supplementary material for: Calcium Improves Germination and Growth of Sorghum bicolor Seedlings under Salt Stress
Source: Plants (Basel). 2020 Jun 10;9(6):730. doi: 10.3390/plants9060730 (PMC7356090; doi:10.3390/plants9060730)
Supplement: Supplementary file 1 [file plants-09-00730-s001.pdf]

plants

Supplementary data

2.1.2 Mean germination time

Mean germination time is a measure of the time it takes for the seed to germinate, focusing on the day at which most seeds have germinated [33]. Mean germination time decreased significantly ($p \leq 0.001$) for seedlings under 300 mM NaCl (Table S1). Application of Ca^{2+} had no significant effect on the mean germination time of control seedlings (Table S1), but 35 mM Ca^{2+} significantly ($p \leq 0.05$) decreased the mean germination time of seedlings under 300 mM NaCl by 0.65-fold (Table S1).

2.1.3 Germination index

The germination index is a parameter that combines the percentage and time of germination, thus the faster a seed lot has germinated, the higher the germination index [33]. A steady decrease in germination index was observed when seedlings were treated with NaCl only (Table S1). NaCl, in particular 300 mM, slowed germination significantly ($p \leq 0.001$), resulting in a germination index of 95.66 as compared to 136 of control seedlings. Ca^{2+} had no significant effect on the germination index of control seedlings (Table S1). Treatment of 300 mM NaCl-stressed seedlings with 5 mM Ca^{2+} significantly ($p \leq 0.01$) increased the germination index (108.25) whereas at 15 mM and 35 mM Ca^{2+}, the germination index was significantly ($p \leq 0.05$) decreased to 85.4 and 77.83 respectively (Table S1).

2.1.4 Total germination

Sodium chloride slightly affected total germination of sorghum seedlings as compared to control seedlings (without added NaCl). Application of 5 mM Ca^{2+} significantly ($p \leq 0.05$) improved the total germination of seedlings under 300 mM NaCl by ~7% whereas 15 and 35 mM Ca^{2+} showed inhibitory effects (Table S1).

Table S1. Effect of Ca^{2+} on the germination attributes of sorghum seedlings in the absence (0 mM) and presence of NaCl (200 and 300 mM). Data represented are mean ± S.D.

Ca^{2+} (mM)	NaCl (mM)	Mean germination time	Germination index	Total germination
0	0	12.59 ± 0.74	136.15 ± 7.70	98.25 ± 3.50
	200	11.28 ± 1.29	120.58 ± 2.23	94.25 ± 4.92
	300	7.92 ± 1.02	95.66 ± 3.93	91.50 ± 1.73
5	0	12.96 ± 0.00	140.00 ± 0.00	100.00 ± 0.00
	200	11.49 ± 1.84	125.33 ± 5.42	95.00 ± 5.77
	300	9.41 ± 1.36	108.25 ± 4.64**	98.25 ± 1.73
15	0	12.96 ± 0.00	140.00 ± 0.00	100.00 ± 0.00
	200	11.22 ± 1.50	124.41 ± 2.38	98.25 ± 3.50
	300	6.92 ± 0.76	85.4 ± 4.80*	83.50 ± 4.04
35	0	12.96 ± 0.00	140.00 ± 0.00	100.00 ± 0.00
	200	9.59 ± 2.29	109.17 ± 5.98**	93.25 ± 4.72
	300	5.19 ± 1.62	77.83 ± 4.26***	84.00 ± 4.24

(* ,** and ***) indicate significant differences at $p \leq 0.05$, $p \leq 0.01$ and $p \leq 0.001$ respectively.

Table S2: Effect of NaCl and Ca^{2+} on the fresh and dry weights of sorghum seedlings in the absence (0 mM) and presence of (200 and 300 mM) NaCl. Data represented are mean ± S.D.

$CaCl_2$ (mM)	NaCl (mM)	Fresh weight	Dry weight
0	0	0.57 ± 0.07	0.14 ± 0.09
	200	0.33 ±0.07***	0.11 ± 0.06
	300	0.29 ±0.07***	0.14 ± 0.08
5	0	0.52 ± 0.17	0.15 ± 0.02
	200	0.33 ± 0.08	0.11 ± 0.06
	300	0.27 ± 0.09	0.11 ± 0.07
15	0	0.62 ± 0.13	0.14 ± 0.03
	200	0.31 ± 0.06	0.10 ± 0.08
	300	0.27 ± 0.06	0.12 ± 0.07
35	0	0.57 ± 0.07	0.16 ± 0.01
	200	0.33 ± 0.09	0.11 ± 0.07
	300	0.26 ± 0.07	0.12 ± 0.06

(***) indicate significant differences at $p \leq 0.001$ respectively.

Table S3. Overall ion content measured by the SEM-Energy dispersive X-ray (EDX) spectroscopy in sorghum seedlings.

Element	0 mM NaCl Wt%	0 mM NaCl Wt sigma	0 mM NaCl At %	5 mM Ca^{2+} Wt%	5 mM Ca^{2+} Wt Sigma	5 mM Ca^{2+} At%	300 mM NaCl Wt%	300 mM NaCl Wt Sigma	300 mM NaCl At%	300 mM NaCl +5 mM Ca^{2+} Wt%	300 mM NaCl +5 mM Ca^{2+} Wt Sigma	300 mM NaCl +5 mM Ca^{2+} At%
C	65,66	0.58	72.58	58,43	0.57	65.54	70,24	0.5	78.52	51.69	1.13	61.59
O	31.78	0.57	26.37	40.32	0.57	33.96	21.03	0.45	17.65	32.6	0.8	29.16
Na	0.18	0.06	0.11	-	-	-	2.43	0.08	1.42	2.89	0.11	1.8
Mg	0.21	0.05	0.12	0.11	0.04	0.06	0.11	0.03	0.06	0.15	0.04	0.09
P	0.76	0.06	0.33	0.37	0.05	0.16	0.32	0.03	0.05	0.22	0.05	0.1
S	0.24	0.04	0.1	0.17	0.04	0.07	Si (0.1)	Si (0.03)	Si (0.05)	Si (0.75)	Si (0.05)	Si (0.35)
Cl	0.14	0.04	0.05	0.12	0.04	0.05	5	0.1	1.89	5.84	0.15	2.36
K	1.02	0.07	0.35	0.37	0.05	0.13	0.76	0.05	0.26	1.86	0.07	0.68
Ca	-	-	-	0.1	0.04	0.03	-	-	-	0.3	0.04	0.11
**Total**	**100**		**100**	**100**		**100**	**100**		**100**	**100**		**100**

Weight % = Wt%, Weight Sigma= Wt Sigma and Atomic % = At%.

Table S4. Accession numbers and primer sequence of genes used for gene expression

Target gene	Forward primer	Reverse primer	Accession number
SbAPX2	5′-AGTCGTGGCAGTTGAGGTAA-3′	5′-ATCCTTGTGGCATCTTCCCA-3′	XM_002463406.2
SbCAT3	5′-GGTTCGCCGTCAAGTTCTAC-3′	5′-AAGAAGGTGTGGAGGCTCTC-3′	XM_021460018.1
SbSOS1	5′-ACACGGGAGAGAGAGAGAGT-3′	5′-TCCAGCTCCAAGTTTGCCTA-3′	XM_002443629.2
Vacuolar SbNHX2	5′-ACTACTGGCGCAAGTTCGAT-3′	5′-TGTCGGCAACACAAAAACAT-3′	XM_002461123.2

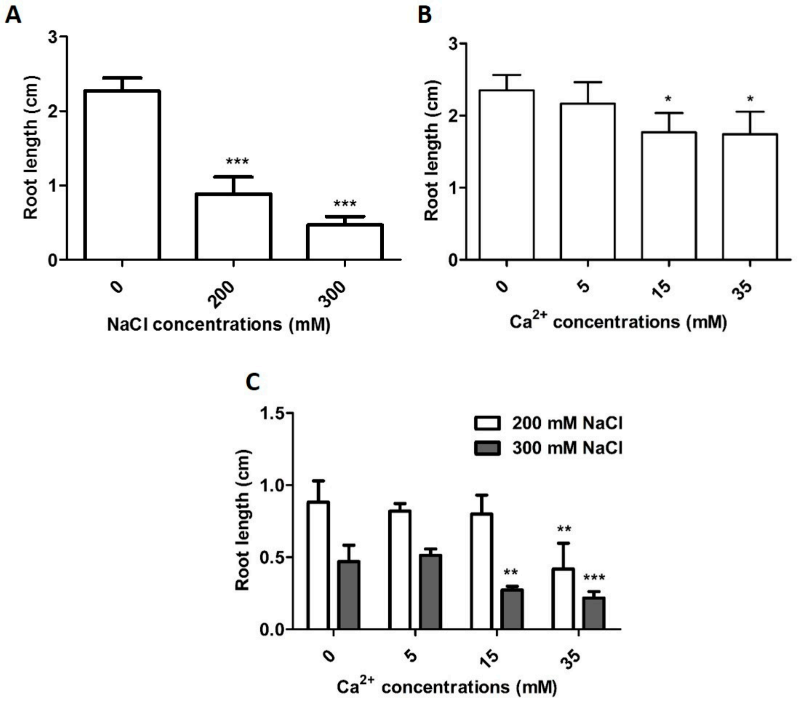

Figure S1: Effect of salt and Ca^{2+} on root and shoot length of sorghum seedlings measured at day 3. (A) Root and shoot length of sorghum seedlings in the presence of different NaCl only. (B-C) Seedlings under different NaCl and Ca^{2+} (5, 15 and 35 mM) concentrations, (B) 0 mM NaCl, (C) 200 mM NaCl and 300 mM NaCl. Error bars represent the S.D calculated from three biological replicates. Statistical significance between control and treated plants were determined using two-way ANOVA conducted on GraphPad Prism 8.4.2, shown as ** = $p \leq 0.01$, and * = $p \leq 0.05$ according to the Bonferroni's multiple comparison test.
